# Deformation imaging to assess global and regional effects of cardiac regenerative therapy in ischaemic heart disease: A systematic review

**DOI:** 10.1002/term.2937

**Published:** 2019-09-01

**Authors:** Bas R. van Klarenbosch, Steven A.J. Chamuleau, Arco J. Teske

**Affiliations:** ^1^ Department of Cardiology University Medical Center Utrecht Utrecht The Netherlands

**Keywords:** 2D speckle tracking, coronary artery disease, deformation imaging, echocardiography, left ventricular ejection fraction, myocardial infarction, stem cells, strain

## Abstract

Currently, left ventricular ejection fraction (LVEF) is the most common endpoint in cardiovascular stem cell therapy research. However, this global measure of cardiac function might not be suitable to detect the regional effects sorted by this therapy and is hampered by high operator variability and loading dependency. Deformation imaging might be more accurate in detecting potential regional functional improvements by cardiac regenerative therapy. The aim of this systematic review is to provide a comprehensive overview of current literature on the value of deformation imaging in cardiac regenerative therapy. A systematic review of current literature available on PubMed, Embase, and Cochrane databases was performed regarding both animal and patient studies in which deformation imaging was used to study cardiac cell therapy. After critical appraisal, outcomes regarding study design, type of cell therapy, procedural characteristics, outcome measure, method for measuring strain, and efficacy on both LVEF and deformation parameters were depicted. A total of 30 studies, 15 preclinical and 15 clinical, were included for analysis. Deformation outcomes improved significantly in 14 out of 15 preclinical studies and in 10 out of 15 clinical studies, whereas LVEF improved in 12 and 4 articles, respectively. Study designs and used deformation outcomes varied significantly among the included papers. Six studies found a positive effect on deformation outcomes without LVEF improvement. Hence, deformation imaging seems at least equal, and perhaps superior, to LVEF measurement in the assessment of cardiac regenerative therapy. However, strategies varied substantially and call for a standardized approach.

**Abbreviations**
2DSTE: two‐dimensional speckle tracking echocardiography
AMI: acute myocardial infarction
GLS: global longitudinal strain
ICMP: ischaemic cardiomyopathy
LVEF: left ventricular ejection fraction
MR: magnetic resonance
MSC: mesenchymal stem cell
SR: strain rate
TDI: tissue Doppler imaging

## 1 INTRODUCTION

Ischaemic heart disease concerns an important health issue in modern‐day medicine. Reperfusion of ischaemic myocardium by catheter‐based and surgical treatment has proven to be successful in both treatment of acute myocardial infarction (AMI) in order to limit infarct size and chronic ischaemic cardiomyopathy (ICMP) in order to protect the heart from further deterioration (Windecker et al., 2014). Because the regenerative capacity of the human heart is intrinsically limited, ischaemic left ventricular (LV) dysfunction currently is a chronic condition. The ability to regenerate infarcted myocardium can be seen as the holy grail within cardiovascular research. Since 2002, when the first clinical trial (Strauer et al., 2002) on the effect of cell therapy on the failing heart was published, clinical research makes effort into regenerating the damaged myocardium (Fernández‐Avilés et al., 2017). Unfortunately, myocardial regeneration has not found its way into the routine clinical setting. However, transfer of progenitor cells into infarcted myocardium has shown to sort a positive, albeit modest, effect on cardiac function (Afzal et al., 2015). The most commonly used functional outcome measure in cardiac regenerative research is the LV ejection fraction (LVEF) as measured by echo or magnetic resonance (MR) imaging, which is easy to perform and effective for measuring global cardiac function changes. In the clinical setting, LVEF has been shown to be a powerful predictor of outcome (Møller et al., 2006; Pocock et al., 2006; Solomon et al., 2005). Notwithstanding these advantages, the technique is hampered by high interoperator and intraoperator variability and is dependent on loading conditions (Dorosz, Lezotte, Weitzenkamp, Allen, & Salcedo, 2012; Wood, Choy, Nanda, & Becher, 2014). Furthermore, LVEF measurement may be less suitable to detect regional function changes potentially sorted by cardiac regenerative therapy. Keeping the high operator variability in mind, LVEF is arguably a suboptimal endpoint.

Cardiac deformation, expressed as strain (ε), is defined as the magnitude of deformation of a segment of myocardium during a cardiac cycle relative to the original length of this segment and is expressed as a percentage. It can be measured using tissue Doppler echocardiography (TDI), two‐dimensional speckle tracking echocardiography (2DSTE), and MR tagging and tracking. Deformation is measured in either direction in which the myocardium deforms: longitudinal (shortening), circumferential (shortening), and radial (thickening; Teske et al., 2007). Peak systolic strain correlates well with LVEF but, unlike LVEF, is more likely to detect regional attenuations of cardiac function (Lima et al., 2017; Weidemann et al., 2002). Deformation imaging is a noninvasive, quantitative, largely operator independent, and therefore reproducible and objective means of measuring both global and regional cardiac function, which at least in the longitudinal direction seems to outperform standard LVEF measurement regarding feasibility, accuracy, and test–retest and interoperator variability (Barbier et al., 2015; Mirea et al., 2018). It is therefore a promising new technique, especially in the field of cardiac regenerative research. However, the additional value of this technique compared with current endpoint measurement within this field is currently inadequately understood.

We hypothesize that deformation imaging will be effective in detecting the potential effects in regional cardiac function sorted by cardiac stem cell therapy. Hence, efficacy of cell therapy can be assessed more precisely. The aim of this systematic review is to identify the added value of strain analysis over standard echocardiography in cardiac regenerative therapy for ischaemic heart disease by providing a comprehensive overview of current literature on cardiac stem cell therapy in which deformation endpoints were used as outcome measures.

## 2 METHODS

### 2.1 Search strategy

According to Preferred Reporting Items for Systematic review and Meta‐Analyses guidelines (Moher, Liberati, Tetzlaff, & Altman, 2009), a systematic search was performed on PubMed, Embase, and Cochrane databases. The search consisted of a combination of three main key terms: “stem cell,” “strain,” and “ischaemia,” with their respective synonyms and related terms (Appendix S1). Using Covidence systematic review software (Veritas Health Innovation, Melbourne, Australia), duplicates were removed. Two authors (B. R. v. K. and A. J. T.) separately assessed each of the identified records for eligibility based on title and abstract. Disagreements were discussed, and consensus was reached, after which the remaining publications were independently assessed on full text by the stated inclusion and exclusion criteria. In addition, references from the identified publications were manually screened in order to identify relevant articles missing from our search.

### 2.2 Inclusion and exclusion criteria

Inclusion criteria were the use of stem cell therapy in ischaemic heart disease, both acute and chronic, and deformation outcomes in either human or large or small animal studies. Exclusion criteria were no (a) full text available; (b) language other than English/Dutch; (c) study design other than randomized controlled trial, randomized trial, case–control study, or cohort study; (d) ex vivo studies; (e) nonischaemic cardiac disease; (f) the use of surgically placed sheets as a delivery method because this is inherently expected to alter deformation parameters; (g) growth factor therapy and intervention; and (h) assessment of diastolic deformation parameters only.

### 2.3 Study characteristics and quality assessment

Of the included articles, the following data on study characteristics were collected: study design (randomized controlled trial, randomized trial, case–control study, and cohort study), population size, setting (acute infarction defined as infusion within a week of myocardial infarction or during the same hospitalization as opposed to ischaemic cardiomyopathy, defined as diminished LVEF ≤ 45% at least 4 weeks after myocardial infarction), and model used (murine, canine, or porcine). The cell type used (e.g., bone marrow‐derived stem cells, mesenchymal stem cells, cardiac stem cells, umbilical cord‐derived stem cells, and pluripotent stem cells) and origin of these cells (either autologous or allogeneic) were identified, as well as the used delivery method (intramyocardial delivery either surgical or percutaneous, intracoronary delivery, and retrograde coronary sinus infusion). As for outcome measures, we assessed the deformation imaging modality utilized (TDI, 2DSTE, MR tagging, or MR feature tracking) and the regional deformation outcomes (regional strain in longitudinal, circumferential, or radial direction), global deformation outcomes (global longitudinal strain, global circumferential strain, or global radial strain), strain rate (SR) outcomes (in either the longitudinal or radial direction), and LVEF measurements. When possible, within‐group changes of the above‐mentioned outcome parameters were derived.

Using the Cochrane risk of bias tool (Higgins et al., 2011), individual studies were assessed for quality. Because data were not pooled, no data synthesis and statistical analyses were performed. In order to provide results in a comprehensive manner, absolute improvement in longitudinal or circumferential strain was depicted as a positive numeral.

## 3 RESULTS

### 3.1 Search results

**Figure 1:**
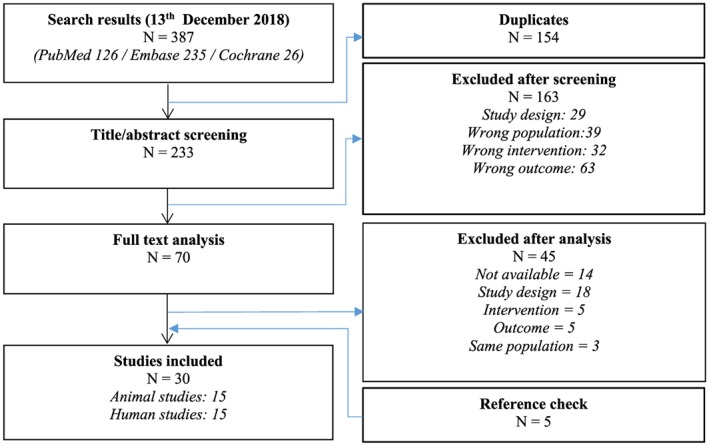
Flow chart of search results [Colour figure can be viewed at http://wileyonlinelibrary.com]

The final search (Figure 1) was performed on December 13, 2018. A total of 126 papers were identified from PubMed database, 235 papers were identified from Embase database, and 26 papers were identified from Cochrane database. After removal of 154 duplicates, 233 titles and abstracts were screened. After exclusion of 163 papers based on title and abstract, 70 papers were selected for full‐text screening. An additional 45 papers were excluded, after which five papers were included after checking cross‐references. Finally, a total of 30 (Amado et al., 2006; Beitnes et al., 2011; Bhatti et al., 2013; Bonios et al., 2011; Cai et al., 2015; Chen et al., 2015; Duran et al., 2013; Heldman et al., 2014; Herbots et al., 2009; Hopp et al., 2011; Jaussaud et al., 2013; Karantalis et al., 2015; Karatasakis et al., 2010; Lebrun et al., 2006; Malliaras et al., 2014; Miao et al., 2014; Nasseri et al., 2009; Nasseri et al., 2014; Plewka et al., 2009; Qi et al., 2015; Quevedo et al., 2009; Rickers et al., 2004; Schneider et al., 2008; Schuleri et al., 2008; Schuleri et al., 2009; Sun et al., 2016; Van Ramshorst et al., 2009; Van Ramshorst et al., 2011; Williams et al., 2011; Yamada et al., 2013) papers were included, of which 15 papers were based on preclinical research and 15 papers were based on clinical research.

### 3.2 Quality assessment and study characteristics

Quality of the included studies was assessed using the Cochrane risk of bias tool (Higgins et al., 2011). Results are shown in Table 1. Five of the included papers, all of these performed in the clinical setting, were double‐blind placebo‐controlled randomized trials where a risk of bias could not be identified. In the remaining 25 studies, a risk of one or more types of bias could not be excluded.

**Table 1:** Quality assessment according to Cochrane risk of bias tool (Higgins et al., 2011)

Study	Study design	Selection bias		Performance bias	Detection bias	Attrition bias	Reporting bias	Other bias
		ASM	ACM					
Preclinical studies								
Amado et al. (2006)	RCT	+	+	‐	?	+	+	+
Bonios et al. (2011)	RCT	+	+	?	?	+	+	+
Cai et al. (2015)	RCT	+	+	?	+	+	+	+
Chen et al. (2015)	RCT	+	+	‐	?	+	‐	+
Duran et al. (2013)	RCT	+	+	‐	+	+	‐	+
Jaussaud et al. (2013)	RCT	+	+	?	+	+	‐	+
Karantalis et al. (2015)	RCT	+	+	?	?	+	+	+
Miao et al. (2014)	RCT	+	+	?	?	+	+	+
Quevedo et al. (2009)	RCT	+	+	?	?	?	‐	+
Rickers et al. (2004)	RCT	+	+	?	?	?	‐	+
Schneider et al. (2008)	RCT	+	+	?	+	+	‐	+
Schuleri et al. (2008)	RCT	+	+	?	?	+	+	+
Schuleri et al. (2009)	RCT	+	+	+	?	+	+	+
Sun et al. (2016)	RCT	+	+	?	?	+	+	+
Yamada et al. (2013)	RCT	+	+	?	+	‐	‐	+
Clinical studies								
Beitnes et al. (2011)	RCT	+	+	‐	+	+	+	+
Bhatti et al. (2013)	RCT	+	+	‐	+	‐	+	+
Heldman et al. (2014)	RCT	+	+	+	+	+	+	+
Herbots et al. (2009)	RCT	+	+	+	+	+	+	+
Hopp et al. (2011)	RCT	+	+	‐	+	+	+	+
Karatasakis et al. (2010)	OCS	‐	‐	‐	‐	+	+	+
Lebrun et al. (2006)	OCS	‐	‐	‐	‐	+	+	+
Malliaras et al. (2014)	RCT	+	+	‐	+	+	+	+
Nasseri et al. (2009)	OCS	‐	‐	‐	‐	+	+	+
Nasseri et al. (2014)	RCT	+	+	+	+	+	+	+
Plewka et al. (2009)	RCT	+	+	‐	?	+	‐	+
Qi et al. (2015)	RCT	+	+	+	+	+	+	+
Williams et al. (2011)	RT	?	‐	‐	?	+	+	+
Van Ramshorst et al. (2009)	CCS	‐	‐	‐	+	+	+	+
Van Ramshorst et al. (2011)	RCT	+	+	+	+	+	+	+

Abbreviations: +, no risk of bias assumed; ‐, possible risk of bias; ?, not reported; CCS, case–control study; OCS, observational cohort study; RCT, randomized controlled trial; RT, randomized trial.

### 3.3 Preclinical study results

Results are shown in Table 2. The following paragraph portrays the heterogeneity of the included articles, due to which pooled analysis or assessment was not feasible. Of the 15 identified papers, seven were based on a porcine model, six on a murine model, and two on a canine model. AMI was studied in 11 papers, the remaining studied animals with ICMP. Sample sizes ranged from 10 to 184 subjects. A total of nine different cell products were used, mesenchymal stem cells (MSCs) being the most common studied cell type. Only three papers use autologous cell types; intramuscular delivery of stem cells was the most commonly used delivery method. Strain was measured using 2DSTE in seven papers, using longitudinal, radial, or circumferential strain as an outcome measure. Four of these papers reported both global and regional deformation outcomes; one paper mentioned both global strain and SR, and the other two papers used SR respectively regional strain only. MR tagging was used in eight papers; all of these were using regional circumferential strain as outcome measure. One paper used TDI, reporting regional radial strain and radial SR. Three papers performed SR analysis. All but one paper reported a statistically significant effect of cell therapy on deformation parameters. Of these, 11 papers also demonstrated a significant positive effect of cell therapy on LVEF. One of these papers, however, only showed a significant effect on regional radial strain, whereas no significant effect on global longitudinal, circumferential, and radial strain was seen. Two papers identified significant changes in regional deformation, whereas LVEF and global strain were not affected by cell therapy or were not reported.

**Table 2:** Studies reporting effect of stem cell therapy on deformation parameters in a preclinical setting

Study	Model	*N*	Cell type	Cell origin	Delivery method	Imaging modality	Endpoint			Effect			Effect LVEF
							Reg	Glo	SR	Reg	Glo	SR	
No significant effect of stem cell therapy on deformation parameters, no significant effect on LVEF													
Sun et al. (2016)	Canine, AMI	28	MSC	NR	RCSI	2DSTE	ɛL	GLS GCS GRS	‐	=	= = =	‐	=
Significant effect of stem cell therapy on deformation parameters, no significant effect on LVEF/LVEF not reported													
Miao et al. (2014)	Murine, AMI	24	MSC	Allo	IM	2DSTE	ɛL ɛC ɛR	GLS GCS GRS	‐	↑ ↑ ↑	= = =	‐	=
Rickers et al. (2004)	Canine, AMI	10	NR	Allo	IM	MR tag	ɛC	‐	‐	↑	‐	‐	‐
Significant effect of stem cell therapy on deformation parameters, significant effect on LVEF													
Amado et al. (2006)	Porcine, AMI	14	MSC	Allo	IM	MR tag	ɛC	‐	‐	↑	‐	‐	↑
Bonios et al. (2011)	Murine, AMI	14	CDC	Allo	IM	2DSTE	ɛC	GCS	‐	↑	↑	‐	↑
Cai et al. (2015)	Murine, AMI	41	H‐CSC	Allo	IM	2DSTE	‐	‐	dɛL/dt	‐	‐	↑	↑
Chen et al. (2015)	Murine, AMI	12	UCBSC	Allo	IM	MR tag	ɛC ɛR	‐	‐	↑ ↑	‐	‐	↑
Duran et al. (2013)	Murine, AMI	184	CBSC CSC	Allo	IM	2DSTE	‐	GLS GRS	dɛL/dt dɛR/dt	‐	↑ ↑	↑ ↑	↑
Jaussaud et al. (2013)	Porcine, iCMP	18	BM‐MSC	Allo	IM	2DSTE	ɛL	‐	‐	↑	‐	‐	↑
Karantalis et al. (2015)	Porcine, ICMP	28	CSC + MSC MSC	Allo	IM	MR tag	ɛC	‐	‐	↑ ↑	‐	‐	↑ =
Quevedo et al. (2009)	Porcine, ICMP	10	MSC	Allo	IM	MR tag	ɛC	‐	‐	↑	‐	‐	↑
Schneider et al. (2008)	Porcine, AMI	23	MSC MSC mBMC	Auto Allo Allo	IM	TDI	ɛR	‐	dɛR/dt	↑ ↑ ↑	‐	↑ ↑ ↑	↑ ↑ ↑
Schuleri et al. (2008)	Porcine, AMI	21	MSC	Auto	IM	MR tag	ɛC	‐	‐	↑	‐	‐	↑
Schuleri et al. (2009)	Porcine, ICMP	15	MSC	Auto	IM	MR tag	ɛC	‐	‐	↑	‐	‐	↑
Yamada et al. (2013)	Murine, AMI	12	IPSC	Allo	IM	2DSTE	ɛR	GLS GCS GRS	‐	↑	= = =	‐	↑

Abbreviations: ‐, not reported; =, no significant effect; ↑, significant positive effect; 2DSTE, two‐dimensional speckle tracking imaging; Allo, allogeneic; AMI, acute myocardial infarction; Auto, autologous; BM‐MSC, bone marrow‐derived mesenchymal stem cell; CBSC, cortical bone stem cell; CDC, cardiosphere‐derived stem cell; dɛL/dt, longitudinal strain rated; ɛC, circumferential strain; ɛL, longitudinal strain; ɛR, radial strain; ɛR/dt, radial strain rate; GCS, global circumferential strain; Glo, global; GLS, global longitudinal strain; GRS, global radial strain; H‐CSC, human cardiac stem cell; ICMP, ischaemic cardiomyopathy; IM, intramuscular; IPSC, induced pluripotent stem cell; LVEF, left ventricular ejection fraction; mBMC, mononuclear bone marrow cell; MR tag, magnetic resonance tagging; MSC, mesenchymal stem cell; *N*, sample size; NR, not reported; RCSI, retrograde coronary sinus infusion; Reg, regional; SR, strain rate; TDI, tissue Doppler imaging; UCBSC, umbilical cord blood stem cell.

Sun et al. (2016) report a nonsignificant absolute increase of 2.5% and 3.6% respectively in both global and regional deformation outcomes for MSC‐treated subjects, compared with 0.66% and 0.69% for saline‐treated controls. LVEF improved nonsignificantly by 1.98% in controls and decreased nonsignificantly by 0.06% in MSC‐treated subjects. The two papers reporting a significant effect of cardiac stem cell therapy on deformation but not on LVEF measurement did not allow for within‐group analysis. In the studies with a significant effect of cell therapy on both deformation and LVEF, which allowed for within‐group analysis, absolute regional strain improvement ranged from 1.1% to 6.3% (Amado et al., 2006; Bonios et al., 2011; Karantalis et al., 2015; Schuleri et al., 2008; Schuleri et al., 2009), compared with −0.6% to 1.3% for control groups. LVEF improvement ranged from 6.9% to 29.8% (Bonios et al., 2011; Karantalis et al., 2015; Schneider et al., 2008; Schuleri et al., 2008; Yamada et al., 2013) for intervention subjects, compared with −7.6% to 2.5% for control subjects.

### 3.4 Clinical study results

Results are shown in Table 3. Similar to preclinical study results, identified articles were markedly heterogeneous in their study design, making the results unsuitable for pooled analysis. Of the 15 identified papers, 10 papers studied patients with ICMP, whereas five studies were carried out in patients with AMI. Sample sizes ranged from eight to 100 patients. The majority of papers studied bone marrow‐derived cells (of which eight studied mononuclear bone marrow cells), whereas three papers studied MSCs, and one paper studied cardiosphere‐derived stem cells. Six papers delivered the cells intramyocardially, and nine performed intracoronary cell infusion. Seven papers measured strain by 2DSTE, whereas four papers determined strain by TDI. MR‐derived strain was reported in five papers, four of which using MR tagging and one using feature tracking. All but one paper measured regional deformation endpoints, either longitudinal or circumferential. Four papers additionally demonstrated global deformation outcomes, as did four papers additionally show longitudinal SR. One paper expressed global longitudinal strain only.

**Table 3:** Studies reporting effect of stem cell therapy on deformation parameters in a clinical setting

Study	Setting	*N*	Cell type	Delivery method	Imaging modality	Endpoint			Effect			Effect LVEF
						Reg	Glo	SR	Reg	Glo	SR	
No significant effect of stem cell therapy on deformation parameters, no significant effect on LVEF												
Beitnes et al. (2011)	AMI	100	mBMC	IC	2DSTE	ɛL	GLS	‐	=	=	‐	=
Bhatti et al. (2013)	AMI	23	BMC	IC	MR FT	ɛC	GCS	‐	=	=	‐	=
Lebrun et al. (2006)	ICMP	11	mBMC	IC	TDI	ɛL	‐	dɛL/dt	=	‐	=	=
Nasseri et al. (2014)	ICMP (pre‐CABG)	52	BMC	IM	2DSTE	ɛL	‐	‐	=	‐	‐	=
Significant effect of stem cell therapy on deformation parameters, no significant effect on LVEF/LVEF not reported												
Heldman et al. (2014)	ICMP	65	mBMC MSC	IM	MR tag	ɛC	‐	‐	↑ ↑	‐	‐	= =
Herbots et al. (2009)	AMI	67	BMC	IC	TDI	ɛL	‐	dɛL/dt	↑	‐	↑	
Hopp et al. (2011)	AMI	28	mBMC	IC	MR tag	ɛC	GCS	‐	↓	↓	‐	=
Karatasakis et al. (2010)	ICMP	10	MSC	IC	TDI	ɛL	‐	dɛL/dt	↑	‐	↑	=
Malliaras et al. (2014)	ICMP	25	CDC	IC	MR tag	ɛC	‐	‐	↑	‐	‐	=
Nasseri et al. (2009)	ICMP	12	BMC	IM	2DSTE	ɛL	‐	‐	↑	‐	‐	‐
Williams et al. (2011)	ICMP	8	mBMC BM‐MSC	IM	MR tag	ɛC	‐	‐	↑	‐	‐	=
Significant effect of stem cell therapy on deformation parameters, significant effect on LVEF												
Plewka et al. (2009)	AMI	60	BMC	IC	2DSTE	ɛL	‐	‐	↑	‐	‐	↑
Qi et al. (2015)	ICMP (pre‐CABG)	42	mBMC	IC (graft)	2DSTE	ɛL ɛC	GLS GCS	‐	= =	↑ =	‐	↑
Van Ramshorst et al. (2009)	ICMP	24	mBMC	IM	2DSTE	‐	GLS	‐	‐	↑	‐	↑
Van Ramshorst et al. (2011)	ICMP	50	mBMC	IM	2DSTE	ɛL	GLS	dɛL/dt	↑	↑	↑	↑

Abbreviations: ‐, not reported; =, no significant effect; ↑, significant positive effect; ↓, significant negative effect; 2DSTE, two‐dimensional speckle tracking imaging; AMI, acute myocardial infarction; BMC, bone marrow cell; BM‐MSC, bone marrow‐derived mesenchymal stem cell; CABG, coronary artery bypass grafting; CDC, cardiosphere‐derived stem cell; dɛC/dt, circumferential strain rate; dɛL/dt, longitudinal strain rate; ɛC, circumferential strain; ɛL, longitudinal strain; Glo, global; GLS, global longitudinal strain; IC, intracoronary; iCMP, ischaemic cardiomyopathy; IM, intramuscular; LVEF, left ventricular ejection fraction; mBMC, mononuclear bone marrow cell; MR FT, magnetic resonance feature tracking; MR tag, magnetic resonance tagging; MSC, mesenchymal stem cell; *N*, sample size; Reg, regional; SR, strain rate; TDI, tissue Doppler imaging.

Four papers (Beitnes et al., 2011; Bhatti et al., 2013; Lebrun et al., 2006; Nasseri et al., 2014) found no significant effect of cell therapy on either regional strain (range −0.2% to 2.6% for the intervention groups compared with 3.0–3.3% for the control groups), global strain (range 2.1–2.5% vs. 0.4–2.4%) or LVEF (range 1.8–2.5% vs. −0.1 to 1.0%), implying no statistically significant beneficial effect of cell therapy on cardiac function. On the contrary, four papers (Plewka et al., 2009; Qi et al., 2015; Van Ramshorst et al., 2009; Van Ramshorst et al., 2011) show a positive effect of cardiac stem cell therapy on both regional (range 1–3.2% for the intervention groups vs. 0–2.3% for the control groups) and global (range 0.8–5.5% vs. −1.2% to 2.3%) deformation outcomes, as well as LVEF measurement (range 4–13% vs. −3% to 6.7%). Qi et al. (2015) showed a beneficial effect of cell therapy on LVEF but only found global longitudinal strain to show a significant positive effect of cell therapy, as opposed to global circumferential strain and regional longitudinal and circumferential strain. Three papers (Plewka et al., 2009; Van Ramshorst et al., 2009; Van Ramshorst et al., 2011) showed regional and global longitudinal strain as a positive outcome measurement of cardiac cell therapy. A total of six papers (Heldman et al., 2014; Herbots et al., 2009; Karatasakis et al., 2010; Malliaras et al., 2014; Nasseri et al., 2009; Williams et al., 2011) found a significant positive effect of cell therapy on regional longitudinal (range 3.7–7.5% for cell treatment groups, no within‐group analyses of control groups supplied) or circumferential (range 2.9–4.9% vs. 0.03% in control patients) strain, whereas an effect on LVEF (range 2.5–5.4% vs. −5.0% to 5.8%) was not detected (*n* = 5) or was not mentioned (*n* = 1). In addition, two of these papers (Herbots et al., 2009; Karatasakis et al., 2010) also found a positive effect of cell therapy on longitudinal SR, and one found a positive effect of cell therapy on global longitudinal strain (Karatasakis et al., 2010). Contrary to expectation, one paper (Hopp et al., 2011) found a significant negative effect of mononuclear bone marrow cell therapy on both regional and global circumferential strain but not on LVEF.

## 4 DISCUSSION

One of the main conclusions that can be drawn from this review is that the use of deformation outcome measures within cardiac cell therapy research is very heterogeneous. The included studies varied substantially in their choice of patient setting or animal model, type of cardiac stem cell, and method of administration. To add to the heterogeneity of our results, the method of deformation measurement (2DSTE, TDI, or MR feature tracking or tagging) and the outcome measure of choice (global or regional strain in the longitudinal, radial, or circumferential direction) varied distinctively between the different included studies. Comparing results of different studies or performing meta‐analyses was therefore not feasible.

Notwithstanding the heterogeneity in deformation imaging within cardiac regenerative medicine, this article provides an insight into the value of strain analysis within this field of research. The results show that in 13 out of 15 preclinical papers and in nine out of 15 clinical papers deformation imaging results followed the results of LVEF outcome measurement, either both showing a significant effect of cardiac cell therapy on LV function or both showing a nonsignificant effect of cardiac cell therapy on LV function. There are no papers that reported a significant effect of cell therapy on LVEF measurement without deformation outcomes doing so. Importantly, and fitting with our main hypothesis, two out of 15 preclinical papers and seven out of 15 clinical papers showed a significant effect of cell therapy on cardiac deformation, whereas LVEF was not improved significantly in these studies. These results advocate a potential added value of deformation imaging within cardiac regenerative medicine for ischaemic heart disease.

Although experience with cardiac stem cell therapy shows a positive effect on cardiac function, underlying mechanisms are not fully elucidated. It is believed to be based on paracrine effects, leading to (a) neovascularization into regions affected by ischaemia (Schuleri et al., 2008), (b) modulation of inflammatory effects (Luger et al., 2017; Van Den Akker, Deddens, Doevendans, & Sluijter, 2013), (c) modulation of ventricular remodelling and extracellular matrix homeostasis (Laflamme, Zbinden, Epstein, & Murry, 2007), and (d) enhancement of cardiac repair by host resident cardiac progenitors (Etzion et al., 2001). Currently, effort is put into optimizing the effect of cardiac regenerative therapy. Indeed, this is reflected by the large heterogeneity in research strategies of the studies included in this article. In addition to these necessary efforts for improving cardiac cell therapy, however, we suggest assessing new outcome measures. This is because effects of cell therapy on global cardiac function as measured by LVEF are limited. In a meta‐analysis assessing the effect of adult bone marrow cardiac cell therapy (Afzal et al., 2015), the authors presented a beneficial effect of bone marrow‐derived cell therapy on LVEF of merely 2.92%. Of importance, the results of papers included in our systematic review are of similar magnitude. Keeping in mind the high interoperator and intraoperator variability and loading dependency of LVEF measurement (Dorosz et al., 2012; Wood et al., 2014), leading to variations of up to 18% for two‐dimensional LVEF measurement by Simpson's rule (Jensen‐Urstad et al., 1998), LVEF is in our opinion not suitable for endpoint measurement in cardiac regenerative medicine in individual cases.

Deformation imaging has already proven its value in, for example, hypertrophic cardiomyopathy (Fuster, Van Der Zee, & Miller, 2009) and valvular heart disease (Casas‐Rojo et al., 2016; Gunjan, Kurien, & Tyagi, 2012), in which deterioration of deformation parameters can be observed before LVEF decreases. This apparent superior sensitivity for changes in cardiac function makes deformation imaging of great potential use within cardiac regenerative medicine. Besides being less operator dependent compared with LVEF, strain analysis allows for assessment of regional cardiac function, making this technique especially suitable for therapies in which a regional effect is expected to be sorted. Regional strain is indeed analysed in all but two included studies in this article, of which seven studies suggest an improved sensitivity to changes in LV function compared with LVEF measurement. There are however no studies reporting on significantly increased global strain, whereas LVEF was not altered. Furthermore, from the results, it is not clear which type of regional strain parameter is best used. As an additional advantage, 2DSTE seems more accurate, also in direct comparison with LVEF measurement (Barbier et al., 2015; Mirea et al., 2018).

The effect of stem cell therapy varies strikingly between preclinical and clinical research: Preclinical papers show a positive effect on deformation parameters more often than clinical studies do. This “translational failure” is a known phenomenon, due to which preclinical results may not always be applicable to the clinical setting. A possible explanation can be found in the risk of bias analysis, showing a higher risk for performance bias, detection bias, and reporting bias in preclinical studies. Also, study models might be more heterogeneous in the patient setting.

## 5 LIMITATIONS

This aim of this study was to investigate whether deformation imaging could provide added value over conventional endpoint measurement in stem cell research. Although evidence is not scarce, and data indeed do suggest added value of deformation imaging, articles that were related to in this study were too heterogeneous in their study design for pooled analysis. Therefore, we could not provide a statistical analysis on deformation imaging as a diagnostic tool over conventional functional imaging. In addition, deformation imaging was not compared with other methods of endpoint measurement, such as myocardial perfusion imaging, so the value compared with these remains to be elucidated. Moreover, the relevance of so‐called surrogate endpoints in stem cell research needs to be addressed. We aimed to investigate deformation imaging as a new surrogate endpoint for stem cell research. However, regardless of the probable additional value of deformation imaging over conventional endpoint measurement, we are unsure how this relates to clinical endpoints such as mortality, hospital admissions, quality of life, or recovery from myocardial infarction. This remains to be studied. To conclude, the conventional echocardiography endpoint used in the included papers was two‐dimensional LVEF, of which diagnostic accuracy is inferior compared with three‐dimensional assessment. Therefore, results might not be applicable to the current situation in which three‐dimensional LVEF assessment is common practice.

## 6 FUTURE PERSPECTIVE

In order to be able to compare deformation outcomes within cardiac regenerative medicine, the methods of performing this should be standardized. We propose to use the “Definitions for a common standard for 2D speckle tracking echocardiography: consensus document of the EACVI/ASE/Industry Task Force to standardize deformation imaging” (Voigt et al., 2015) as a guide for standardized deformation imaging within cardiac regenerative research. This provides guidance into both image acquisition and image processing. Preferably, deformation should be measured using 2DSTE or MR imaging tagging or feature tracking; TDI‐derived strain is arguably too labourious and complicated for common clinical use. Future efforts of this task force might further enhance diagnostic performance of 2DSTE and allow for more interstudy comparison. Future research might provide insight into which the deformation parameters are most sensitive to LV functional change after regenerative therapy. Also, newer strategies such as three‐dimensional speckle tracking echocardiography might provide additional diagnostic accuracy.

## 7 CONCLUSION

Deformation imaging shows to be a promising new endpoint measure in cardiac stem cell research, which is at least equally, and possibly more, effective than LVEF assessment in the detection of changes in cardiac function and may provide research with a more objective measure of response to therapy. However, current use is diverse and needs standardization in order to compare results between studies.

## CONFLICT OF INTEREST

None declared.

## APPENDIX A.

### Search syntax

#### PubMed

(((((((((((Stem cell [title/abstract]) OR stem cells [title/abstract]) OR progenitor [title/abstract]) OR mother cell [title/abstract]) OR mother cells [title/abstract]) OR colony forming [title/abstract]) OR colony‐forming [title/abstract]) OR regeneration [title/abstract]) OR regenerative therapy [title/abstract]) OR bone marrow [title/abstract]))AND (((((((Strain [title/abstract]) OR deformation [title/abstract]) OR regional myocardial function [title/abstract]) OR speckle tracking [title/abstract]) OR tissue Doppler [title/abstract]) OR tissue velocity [title/abstract])) AND ((((((((((((((((((((((((((Myocardial infarction [title/abstract]) OR myocardial infarctions [title/abstract]) OR myocardial infarct [title/abstract]) OR myocardial infarcts [title/abstract]) OR myocardial ischaemia [title/abstract]) OR myocardial ischemia [title/abstract]) OR cardiac ischemia [title/abstract]) OR cardiac ischaemia [title/abstract]) OR cardiovascular stroke [title/abstract]) OR heart attack [title/abstract]) OR heart attacks [title/abstract]) OR coronary [title/abstract]) OR ischemic cardiomyopathy [title/abstract]) OR ischaemic cardiomyopathy [title/abstract]) OR ischemic heart failure [title/abstract]) OR ischaemic heart failure [title/abstract]) OR ischemic heart disease [title/abstract]) OR ischaemic heart disease [title/abstract]) OR CAD [title/abstract]) OR IHD [title/abstract]) OR STEMI [title/abstract]) OR NSTEMI [title/abstract]) OR CHD [title/abstract]) OR AMI [title/abstract]) OR ACS [title/abstract]))

#### EMBASE

(‘stem cell’:ti,ab OR ‘stem cells’:ti,ab OR ‘progenitor’:ti,ab OR ‘mother cell’:ti,ab OR ‘mother cells’:ti,ab OR ‘colony forming’:ti,ab OR ‘colony‐forming’:ti,ab OR ‘regeneration’:ti,ab OR ‘regenerative therapy’:ti,ab OR ‘bone marrow’:ti,ab) AND (‘strain’:ti,ab OR ‘deformation’:ti,ab OR ‘regional myocardial function’:ti,ab OR ‘speckle tracking’:ti,ab OR ‘tissue doppler’:ti,ab OR ‘tissue velocity’:ti,ab) AND (‘myocardial infarction’:ti,ab OR ‘myocardial infarctions’:ti,ab OR ‘myocardial infarct’:ti,ab OR ‘myocardial infarcts’:ti,ab OR ‘myocardial ischaemia’:ti,ab OR ‘myocardial ischemia’:ti,ab OR ‘cardiac ischemia’:ti,ab OR ‘cardiac ischaemia’:ti,ab OR ‘cardiovascular stroke’:ti,ab OR ‘heart attack’:ti,ab OR ‘heart attacks ’:ti,ab OR ‘coronary’:ti,ab OR ‘ischemic cardiomyopathy’:ti,ab OR ‘ischaemic cardiomyopathy’:ti,ab OR ‘ischemic heart failure’:ti,ab OR ‘ischaemic heart failure’:ti,ab OR ‘ischemic heart disease’:ti,ab OR ‘ischaemic heart disease’:ti,ab OR ‘CAD’:ti,ab OR ‘IHD’:ti,ab OR ‘STEMI’:ti,ab OR ‘NSTEMI’:ti,ab OR ‘CHD’:ti,ab OR ‘AMI’:ti,ab OR ‘ACS’:ti,ab)

#### COCHRANE

(“stem cell":ti,ab,kw OR “stem cells":ti,ab,kw OR “progenitor":ti,ab,kw OR “mother cell":ti,ab,kw OR “mother cells":ti,ab,kw OR “colony forming":ti,ab,kw OR “colony‐forming":ti,ab,kw OR “regeneration":ti,ab,kw OR “regenerative therapy":ti,ab,kw OR “bone marrow":ti,ab,kw) AND (“strain":ti,ab,kw OR “deformation":ti,ab,kw OR “regional myocardial function":ti,ab,kw OR “speckle tracking":ti,ab,kw OR “tissue doppler":ti,ab,kw OR “tissue velocity":ti,ab,kw) AND (“myocardial infarction":ti,ab,kw OR “myocardial infarctions":ti,ab,kw OR “myocardial infarct":ti,ab,kw OR “myocardial infarcts":ti,ab,kw OR “myocardial ischaemia":ti,ab,kw OR “myocardial ischemia":ti,ab,kw OR “cardiac ischaemia":ti,ab,kw OR “cardiac ischemia":ti,ab,kw OR “cardiovascular stroke":ti,ab,kw OR “heart attack":ti,ab,kw OR “heart attacks":ti,ab,kw OR “coronary":ti,ab,kw OR “ischemic cardiomyopathy":ti,ab,kw OR “ischaemic cardiomyoapthy":ti,ab,kw OR “ischemic heart failure":ti,ab,kw OR “ischaemic heart failure":ti,ab,kw OR “ischemic heart disease":ti,ab,kw OR “ischaemic heart disease":ti,ab,kw OR “CAD":ti,ab,kw OR “IHD":ti,ab,kw OR “STEMI":ti,ab,kw OR “NSTEMI":ti,ab,kw OR “CHD":ti,ab,kw OR “AMI":ti,ab,kw OR “ACS":ti,ab,kw)
